# Editorial: Plant metabolites in drug discovery: the prism perspective between plant phylogeny, chemical composition, and medicinal efficacy, volume III

**DOI:** 10.3389/fphar.2024.1530039

**Published:** 2024-12-13

**Authors:** Da-Cheng Hao, Yao-Xuan Wang, Richard W. Spjut, Chun-Nian He

**Affiliations:** ^1^ Department of Environment Science and Engineering, Biotechnology Institute, Dalian Jiaotong University, Dalian, China; ^2^ World Botanical Associates Inc., Bakersfield, CA, United States; ^3^ Institute of Medicinal Plant Development, Chinese Academy of Medical Sciences and Peking Union Medical College, Beijing, China

**Keywords:** pharmacophylogeny, phylogenomics, phytometabolite, bioactivity, omics

**FIGURE 1:**
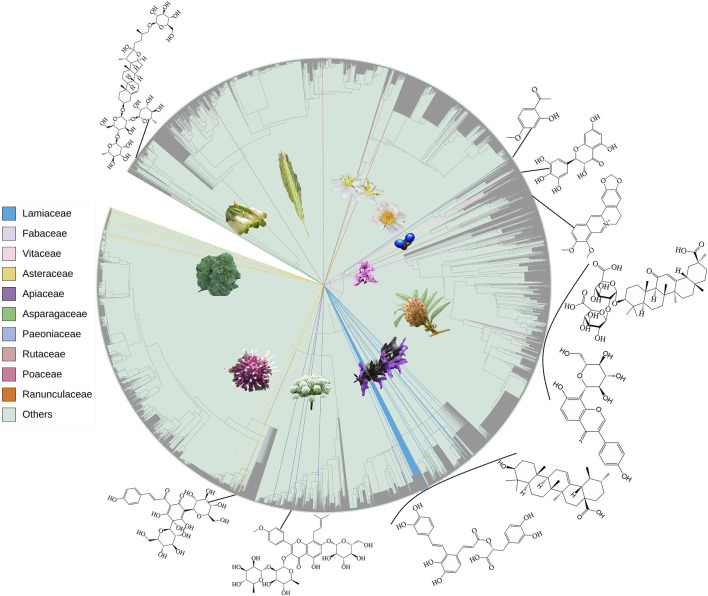
Phylogenetic distribution of botanical taxa, reported in Vol III of this Research Topic, as well as representatives of medicinal phytometabolites therein. Among totally 155 species, 85 genera of 44 families, only 118 species, 49 genera of top ten families are shown.

There is a Chinese saying that goes, “The most profound takes the simpliest form.” The phylogenetically close taxa usually have similar phytometabolite profiles, and taxa that are closely related in chemotaxonomy often have analogous ethnopharmacological uses and similar bioactivities. This is the simple core idea of “Pharmacophylogeny,” and the proposed term “Pharmacophylomics” aims to disentangle the intricate relationships and connectivity between medicinal plant phylogeny, phytochemical constituents and bioactivities/therapeutic utilities based on emerging omics data (Figure 1) (Hao and Xiao, 2023); the genomic, transcriptomic and metabolomic data are very useful to promote pharmaceutical resource discovery and plant-based drug R&D. In recent years, more phytomedicine researchers become familiar with the theory and methods of pharmacophylogeny (Moutouama and Gaoue, 2024), but the research on the simultaneous examination of phylogeny/phylogenomics, chemical constituents, and bioactivity is still limited. Based on the 21 papers published in Volume I and II of this Research Topic (Hao et al., 2023a), volume III further contributed eight enlightening papers on the phylogenomics, metabolomics, network pharmacology, ethnopharmacology and bioactivity of various medicinal species, covering algae (Su et al.), monocot (Bencheikh et al.; Luo et al.), basal eudicot (Bencheikh et al.; Pan et al.), core eudicot (Ding et al.), Lamiids (Liu et al.) and Campanulids (Luo et al.). These works provide rich phytometabolite and bioactivity information for deeper exploration on pharmacophylogeny, facilitating the analysis of distribution patterns of various medicinal compounds and pharmacological activities on the phylogenetic tree (Hao et al., 2024a), the inference of biosynthetic pathways and therapeutic mechanisms of phytometabolites, and the search for alternative/complementary medicine sources.

Fermentation is an important processing technique for phytomedicine in transforming and enhancing the active ingredients of herbal medicine via specific microbial processes (Luo et al.). Thus, fermentation adds another dimension to pharmacophylogeny. Similar fermentation strains when used in species with similar phytometabolites—for generating microbial transformations—expands the use of fermented botanical drugs in the areas anti-cancer, hypolipidemic, antioxidant, antimicrobial, cosmetics, and gut microflora regulation. The fermentation technology also reduces toxic effects of some crude drugs, while enhancing drug efficacy (Pan et al.). The puzzling interaction between microbial strains and crude drugs is another intriguing Research Topic. Managing factors affecting the microbial activities and fermentation process is vital for the successful transformation and efficacy improvement of herbal drugs. For example, by assuming that phylogenetically related bacteria and fungi similarly interact with specific plant compounds, one may predict the similarities and differences in the spectrum of transformation products based on the composition of the microbial community. There is much more work to be done in this area, which is not limited to TCM species. Fermentation research can be conducted on all ethnomedicinal species around the world (Bencheikh et al.; Liu et al.) at the genus level. Molecular phylogeny and metabolomic information are essential to understand medicinal value of each microbial/plant genus in developing alternative medicine. This is also applicable within the food medicine continuum (FMC) in ethnomedicinal plants worldwide. Pharmacophylogeny can aid in expand FMC and medicinal plant resources (Hao and Xiao, 2023), authenticate quality control of herbal medicines, predict the chemicals or bioactive constituents of herbs and identify/quantify chemicals. Reports on phytometabolites and pharmacological properties of algae (Su et al.) are relatively fewer as might be expected by the fewer species number; however, their association with fungi and bacteria in the lichen biome has scarcely been investiaged (Rakotondraibe et al. 2024). In the coming years, pharmacophylogeny and pharmacophylomics can be expected to improve mining of natural products from taxa of various evolutionary levels (Lu and Tang, 2020), refining ethnopharmacology understandings, and therefore advance the sustainable conservation and consumption of natural pharmaceutical resources.

The global medicinal plant diversity is threated by the increasingly intense anthropogenic activities, while phrmacophylogeny and pharmacophylomics help sustainable conservation and utilization of precious phytomedicine resources. Based on the harvest of first two volumes, the volume III (https://www.frontiersin.org/research-topics/62190/plant-metabolites-in-drug-discovery-the-prism-perspective-between-plant-phylogeny-chemical-composition-and-medicinal-efficacy-volume-iii) strives to gain a deeper understanding of phylogeny/evolution, phytometabolites and poly-pharmacology of genera/families of interest. We always advocate to conduct such investigations within the context of pharmacophylogeny and pharmacophylomics, and we are happy to publish such comprehensive work in this volume. The Lamiaceae genus *Dracocephalum*, with more than 30 species, possesses diverse medicinal activities and is traditionally used in Eurasian ethnomedicine (Liu et al.). The geographical distribution, metabolite identification, and bioactivity of *Dracocephalum* species were extensively investigated, but there are debates on the taxonomy of *Dracocephalum* and closely related genera *Hyssopus* and *Lallemantia*, which presents an opportunity for pharmacophylogenetic studies of these medicinal taxa. Liu et al. present a multidimensional view of the geographical distribution, phylogenetics, phytometabolites and chemodiversity, ethnopharmacological uses, and pharmacology of *Dracocephalum*, *Hyssopus*, and *Lallemantia*. The species in the latter two genera are concentrated in southwest Asia and those in *Dracocephalum* are distributed across temperate northern hemisphere. Although all three genera are closely related phylogenetically, the species of *Hyssopus* were intertwined with those of *Dracocephalum* on the phylogenetic tree. Among more than 900 reported phytometabolites of three genera, terpenoids and flavonoids are the most abundant. The newly identified novel metabolites of *Dracocephalum* expand chemical space to be bioprospected. Ethnopharmacologically, these three genera are especially useful in treating respiratory, liver and gall bladder diseases. Phytometabolites of these genera have various bioactivities such as hepatoprotective, anti-inflammation, antimicrobial action, anti-hyperlipidemia, and anti-tumor properties. Integrating phylogenetics and network pharmacology enabled exploring the intricate links between metabolite profiles, traditional efficacy, and modern pharmacology of *Dracocephalum* and its related genera. This study illustrates how to discover potential medicinal value from closely related ethnomedicinal taxonomic groups.

Another endeavor in Vol III is reconstructing the phylogenetic tree of *Glycyrrhiza* and related subfamilies of Fabaceae based on the whole chloroplast (cp) genome sequences (Wu et al.). China has eight species of *Glycyrrhiza* (Chen et al., 2020), which can be classified into two sections based on the presence of glycyrrhizic acid: section *Glycyrrhiza* (*G. uralensis* Fisch., *G. glabra* Linn., *G*. *inflata* Batal., *G. aspera* Pall., *G*. *eglandulosa* X. Y. Li) and section *Pseudoglycyrrhiza* (*G*. *pallidiflora* Maxim., *G*. *squamulosa* Franch., *G*. *yunnanensis* Cheng f. et L. K. Dai ex P. C. Li). The cp genome-based phylogeny confirmed this classification, and suggested that *G. gobica* Grankina and *G. uralensis* clustered together, *G. laxissima* Vassilcz. and *G. aspera* clustered together, and other taxa, which are treated as synonyms of *G. uralensis*, *G. glabra* or *G. aspera* in Flora of China, should be considered as independent species. The North American species *G. lepidota* had a lower content of glycyrrhizic acid and was in another group, indicating that the groups containing glycyrrhizic acid were not monophyletic, i.e., the incongruence between phylogenomics and chemotaxonomy. The cp genome-based phylogeny also revealed distinct intraspecific divergence in 38 *Artemisia annua* strains (Ding et al.). These results are very beneficial for us to fully understand the complexity of medicinal kinship at various taxonomic levels.

It is expected that the metabolomic analyses and phytometabolite content determination could reveal the overall similarity of phytometabolite profiles between medicinally important species, such as *Astragalus membranaceus* (Li et al.), *Dioscorea opposita*, and *Rehmannia glutinosa*, and phylogenetically related species, and both molecular authentication and chemotaxonomy could be used to discriminate them from common adulterants. On the other hand, the R&D of innovative herbal medicine formulas can also benefit from detailed pharmacophylogenetic studies, as closely related species can be added to the medicine formulas, and various combinations of them can be tried to observe changes in therapeutic effects, which may improve the efficacy of existing herbal formulas or expand novel therapeutic approaches. The cp genome is an useful genetic resource for phylogeny and evolution studies at both species and subspecies/population levels, while the interspecific/intraspecific chemodiversity could lead to development of novel clinical utility.

In summary, the greatest truths are the simplest; taxa in sister phylogenetic groups have relatively similar requirements for ecological environment conditions (Hao et al., 2024b), they have closely related genetic features, and are more likely to evolve analogous biosynthetic pathways, therefore their chemical ammunition depot could be more similar, resulting in the global resemblance of bioactivity or therapeutic efficacy (Hao et al., 2024a). However, if we want to condense scientific hypotheses and practical solutions from the complex phenomena and vast amounts of data, hard work is essential. Integrating ecological and evolutionary factors helps to gain a more inclusive understanding of phytochemical changes in changing environments (Hao et al., 2023b). With any luck the papers in Volumes I-III of Research Topic could serve as valuable references and enhance researchers’ cognizance of pharmacophylogeny, and we wish more scholars intentionally leverage pharmacophylomic methods to the conservation, exploration, and utilization of medicinal species.
